# Illness perceptions in people with chronic and disabling non-specific neck pain seeking primary healthcare: a qualitative study

**DOI:** 10.1186/s12891-024-07302-7

**Published:** 2024-02-27

**Authors:** Maaike Kragting, Annelies L. Pool-Goudzwaard, Michel W. Coppieters, Peter B. O’Sullivan, Lennard Voogt

**Affiliations:** 1 Department of Physical Therapy, Research Centre for Health Care Innovations, Rotterdam University of Applied Sciences, Rochussenstraat 198, Rotterdam, 3015 EK The Netherlands; 2 Department of Human Movement Sciences, Faculty of Behavioural and Movement Sciences, Amsterdam Movement Sciences – Program Musculoskeletal Health, Vrije Universiteit Amsterdam, Amsterdam, The Netherlands; 3 Somt University of Physiotherapy, Amersfoort, The Netherlands; 4 School of Health Sciences and Social Work, Menzies Health Institute Queensland, Griffith University, Brisbane and Gold Coast, Australia; 5 School of Allied Health, Curtin University, Bentley, Australia; 6 Body Logic Physiotherapy Clinic, Shenton Park, Australia; 7 Pain in Motion Research Group, Department of Physiotherapy, Human Physiology and Anatomy, Faculty of Physical Education & Physiotherapy, Vrije Universiteit Brussel, Brussels, Belgium

**Keywords:** Beliefs, Pain perceptions, Illness representations, Behaviour, Qualitative research

## Abstract

**Background:**

Illness perceptions can affect the way people with musculoskeletal pain emotionally and behaviorally cope with their health condition. Understanding patients illness perceptions may help facilitate patient-centered care. The purpose of this study was to explore illness perceptions and the origin of those perceptions in people with chronic disabling non-specific neck pain seeking primary care.

**Methods:**

A qualitative study using a deductive and inductive analytical approach was conducted in 20 people with persistent (> 3 months) and disabling (i.e., Neck Disability Index ≥ 15) neck pain. Using a semi-structured format, participants were interviewed about their illness perceptions according to Leventhal’s Common Sense Model. Purposive sampling and member checking were used to secure validity of study results.

**Results:**

Participants reported multiple symptoms, thoughts and emotions related to their neck pain, which continuously required attention and action. They felt trapped within a complex multifactorial problem. Although some participants had a broader biopsychosocial perspective to understand their symptoms, a biomedical perspective was dominant in the labelling of their condition and their way of coping (e.g., limiting load, building strength and resilience, regaining mobility, keep moving and being meaningful). Their perceptions were strongly influenced by information from clinicians. Several participants indicated that they felt uncertain, because the information they received was contradictory or did not match their own experiences.

**Conclusion:**

Most participants reported that understanding their pain was important to them and influenced how they coped with pain. Addressing this ‘sense making process’ is a prerequisite for providing patient-centered care.

**Supplementary Information:**

The online version contains supplementary material available at 10.1186/s12891-024-07302-7.

## Introduction

Health perceptions of people with musculoskeletal pain influence their emotional wellbeing and the way they cope with their health condition [1, 2]. These perceptions can be both barriers and facilitators to regain optimal health [3, 4]. Unhelpful perceptions (e.g., beliefs of tissue damage despite the absence of specific pathology) and negative emotions (e.g., pain-related fear and distress) can negatively influence a person’s health behaviour (e.g., passive coping and/or avoidance of activities) [5]. This can hinder recovery and increase the impact of musculoskeletal pain on people’s lives [1, 6, 7]. Understanding and addressing unhelpful illnessperceptions may therefore be important for healthcare professionals working with people with musculoskeletal pain [4, 8]. There is emerging evidence that illness perceptions and emotional responses are amenable to change through cognitive and behaviourally targeted interventions, enhancing a person’s sense of control over their health condition and improving their long-term functioning [9–11]. Furthermore, it is known that illness perceptions and self-efficacy mediate treatment outcome (e.g., physical functioning and pain intensity) in people with musculoskeletal pain (i.e., low back and shoulder pain) [12–18].

The Common Sense Model proposed by Leventhal [7, 19] is designed to help frame a person’s perceptions, emotions and behavioural responses to their health condition or illness. This model is widely used to explore how ‘sense-making processes’ influence coping [20]. According to the Common Sense Model, when people become ill they try to make sense of their illness by forming cognitive and emotional representations of their condition (i.e., a lay model of illness perceptions). In these representations five themes can be recognised [19]: 1) ‘What do I have?’ (‘identity’ beliefs), 2) ‘How did I get it?’ (‘cause’ beliefs, including provoking factors) [21], 3) ‘How long will it take?’ (‘timeline’ beliefs), 4) ‘What are the consequences?’ (‘consequence’ beliefs), and 5) ‘How can I control it?’ (‘controllability’ beliefs). Later research added the themes ‘Do I understand my illness?’ (coherence) and (‘How do I feel about it?’ (emotional representation) [22]. This representation informs the actions someone takes. Lay perceptions are strongly influenced by previous experiences with pain [10, 23], observed behaviour and/or the information people receive from others (e.g., healthcare professionals, family) [10, 24, 25].

Illness perceptions in people with neck pain have been explored qualitatively in people with acute and chronic whiplash associated disorders (WAD) [10, 26–28]. In these studies, the following main themes influencing recovery were identified: ‘a continuous search for the appropriate strategy to (self)manage symptoms’ [26, 27], ‘expectations of recovery’ [10, 27], ‘self-efficacy beliefs’ [10] and ‘a condition with multiple symptoms’ [26]. These themes underline the importance of ‘sense-making processes’ in people with neck pain, as proposed by the Common Sense Model. This work highlights that people with neck pain are not always provided with an evidence informed explanation for their pain and helpful strategies to resolve it [10, 26, 27, 29]. However, the perceptions in the WAD-group might differ from those in the broader group of people with neck pain [30].

Patients with disabling non-traumatic neck pain are a large group [31, 32] with a high burden of disease [33] and a poor prognosis [34] who continue to seek care [29] and whose condition is often resistant to treatment [35]. To date, qualitative studies conducted in the broader group of people with neck pain have focussed on treatment experiences [29, 36] or symptoms and provoking factors [37]. However, an in-depth exploration of illness perceptions in people with disabling chronic non-specific neck pain (i.e., neck pain without a specific underlying pathology, classified as neck pain grade II [38]), using the Common Sense Model, is lacking. Furthermore, it is unclear how people with disabling neck pain form their illness perceptions and what role healthcare professionals play in the development of their patients’ representation of their neck pain.

This study aimed to explore [1] the illness perceptions of people with disabling chronic non-specific neck pain using the Common Sense Model as a conceptual framework; and [2] how these illness perceptions were formed.

## Materials and methods

A qualitative study was conducted to explore the illness perceptions of people with disabling chronic non-specific neck pain. In qualitative research it is assumed that people give meaning to phenomena and experiences and exchange them in social interactions [39, 40]. Content analysis was used to systematically analyse the beliefs and experiences described in the interviews and identify categories through an iterative process of coding and interpretation [41, 42]. The protocol of this study was approved by the Scientific and Ethical Review Board (VCWE) of the Faculty of Behavioural and Movement Sciences, Vrije Universiteit Amsterdam, The Netherlands (VCWE-2019-127). All participants provided written informed consent prior to participating in the study.

### Participants

The research population consisted of people with moderately or severely disabling neck pain (i.e., Neck Disability Index (NDI) = 15–24 points for moderate disability and ≥ 25 for severe disability [43, 44]). Participants were recruited in the region of Rotterdam by primary care physiotherapists (via flyers in the waiting room or information after a treatment consultation) or via social media posts (Facebook and Twitter). Purposive sampling was employed based on age, sex and duration of neck pain. When people were willing to participate, a research assistant contacted them, explained the study and sent the person both an information letter and the NDI questionnaire. If the person returned a NDI of ≥ 15, the coordinating researcher (MK) contacted the person by telephone to screen for further eligibility, using the following selection criteria: (1) aged > 18; (2) neck pain with no signs of serious pathology (which had to be confirmed later by the treating therapist), but which interfered with daily activities (i.e., neck pain Grade II) [38]; (3) neck pain for a minimum of 3 months; and (4) sufficient command of the Dutch language. People were excluded if they had neck pain with neurological signs (i.e., neck pain Grade III) or neck pain with signs of serious pathology (i.e., neck pain Grade IV) [38]. The number of participants was based on the moment that theoretical saturation was achieved (i.e., no new relevant information was obtained when analysing the information from newly selected participants) [39]. The participants had no previous relationship with the interviewer.

### Questionnaires

Prior to the interview, demographic (sex and age) and neck pain related data (duration of neck pain, pain intensity (Nummeric Pain Rating Scale (NPRS)), physical function (Patient Specific Functional Scale (PSFS)) and NeckPix [45]) and illness perceptions (Brief Illness Perception Questionnaire (BIPQ)) were gathered using questionnaires. Demographic data and scores on the NPRS, NDI, PSFS and Neckpix were used to describe the characteristics of the participants. Information from the PSFS, IPQ and Neckpix were used by the interviewer as prompts to chart their perceptions in more detail.

The NPRS is a simple and valid tool to measure pain intensity on a 11-point scale [46], with higher scores representing higher pain intensity. The NDI is a reliable tool with good to excellent internal consistency to assess the level of disability in people with neck pain and consists of 10 items with six response categories (range 0–5, total score range 0–50), with higher scores representing higher disability [47–50]. The Neckpix is a reliable pictorial scale to measure fear of movement in people with chronic neck pain consisting of a specific set of 10 daily activities on a 11-point NRS scale, with higher scores representing higher fear. This scale has a strong correlation with the Tampa Scale for Kinesiophobia [45], is region-specific and easy to understand due to the use of pictures [51, 52]. The PSFS is the recommended tool in neck pain guidelines that tries to quantify a patient’s disabilities in daily functioning on an 11-point NRS scale [53–55].

The Brief Illness Perception Questionnaire Dutch Language Version (BIPQ-DLV) consists of 8 items (using a 11-points NRS scale) and one open question (i.e., ‘Please list in rank-order the three most important factors that you believe caused your neck pain’) that quantifies the five components of illness representations in Leventhal’s Common Sense Model added by coherence and emotional representations as well [56, 57]. The subscales can be used to clarify the patient’s perspective on his/her health problem and how he/she copes with it [58].

### Interview

A semi-structured interview was used, based on the themes of Leventhal’s Common Sense Model [19]. This model provides the opportunity to thoroughly explore each person’s unique illness perceptions regarding the identity, causes, timeline, consequences, controllability, coherence and emotional representations of their neck pain and their behavioural responses. We used the model as sensitizing concept, to structure the interviews and to maintain focus on the purpose of the study [59], without restricting the participants from telling about their thoughts, by using open-ended questions (see Supplementary file 1 ‘conversation guide’). The interview took place either at the participant’s home, in a quiet room in the physiotherapy clinic, or online (due to Covid) in a secure MS Teams environment.

Each interview was led by one investigator (MK) who is an experienced physiotherapist, lecturer and clinical researcher with more than 25 years of relevant clinical experience, assisted by a research-assistant who monitored use of the conversation guide. This female investigator was trained in interview techniques and succesfully completed formal training in qualitative research. The conversation guide guaranteed that every interview was structured in a similar way and that the same topics were covered. Existing literature was used to develop the conversation guide [27, 28, 60]. The conversation guide was then discussed with three experts in the field of qualitative research. Following the principles of qualitative research as an iterative and reflexive process, the conversation guide changed slightly as a result of experiences during previous interviews [61]. A logbook was used to make field notes about the reason for slightly modifying the questions. Any deviations from the conversation guide were noted.

The interviews were recorded, transcribed, summarized, coded, analysed and interpreted (for a more detailed description see data analysis). Full texts were submitted to the participants for verification (i.e., member check). Any additions or changes that the participants reported were noted.

### Data analysis

Data analysis was conducted by the entire research team, which consisted of experienced lecturers, researchers and clinicians in the field of Physiotherapy and Psychology, who all had experience in conducting and publishing several qualitative studies in the field of musculoskeletal health [62–66]. The transcripts were read and anonymised before being analysed using the Computer Assisted Qualitative Data Analysis Software (version Atlas-ti.8 Windows, Scientific Software Development GmbH, Berlin, Germany). To analyse the data, first, a deductive approach was used to sort the data into the broad categories of the Common Sense Model and maintain alignment with the research questions [59, 67]. Then, an inductive analytical approach was used to make meaning from the data [39, 59]. The following steps were taken:

Step 1: Independent parallel coding [42]. Two researchers (MK, and LV or AP) independently read the first six transcripts in detail and coded the text guided by the main research questions (i.e., open coding [39, 59]): 1) What are the specific illness perceptions of people with disabling chronic non-specific neck pain who are moderately or severely disabled regarding their health condition, and 2) How were these illness perceptions formed? Guided by the data, new categories could emerge [39, 59].

Step 2: Development of categories into a framework [42]. The coding and developed categories were discussed with three members of the research group (MK, LV and AP), which lead to a concept category system. The meaning of each category was described in a codebook. This concept category system was applied to the next eight transcripts (all by MK, and two by LV or AP). A second discussion meeting was organised in which the category system was further refined and definitive ‘main categories’ were established.

Step 3: All the transcripts were read and coded by MK according to the definitive category system. Subsequently the raw data were interpreted [42].

Step 4: Discussion about the interpretations [42]. The interpretation of the data, including relationships between the categories, were discussed in a third and fourth meeting with the abovementioned three members of the research group and a fifth and sixth meeting with all investigators (MK, LV, AP, POS, MC).

Finally, the data were reported using the criteria for reporting qualitative research (COREQ) [68]. Consistent with this qualitative approach it was not the aim to quantify the responses. However, to provide readers with an indication of the frequency of endorsement of each theme we have used the terms ‘unanimous/(almost) all’ (≥ 95% = ≥19 participants); ‘most’ (more than 75% = >15 participants); ‘many’ (50%, nearly 10 participants); ‘some’ (more than 20% = 4–10 participants); ‘several (< 20%)/ a few’ (< 10%) < 4 participants) [69].

## Results

### Participants

The interviews were conducted between November 2019 and December 2020. Saturation was reached with 20 participants. To obtain 20 participants, we had to invite 26 people with neck pain of which 25 people were willing to participate in the study. Four of them did not meet the inclusion criteria because their NDI score was < 15. One person withdrew after the inclusion procedure because participating was considered too burdensome. Twenty participants (15 women, 5 men) completed the questionnaires and the interview (mean (SD) age: 46.8 (12.1) years; median and Inter Quartile Range (Q25-Q75) duration of neck pain: 21.0 (6.0-117.0) months; mean (SD) pain intensity; 5.8 (1.8); median (Q25-Q75) NDI score; 20.0 (16.0-25.8); mean (SD) Neckpix score; 36.3 (27.8)). Please see Table 1 for the specific characteristics of each participant. At the time of the study, 18 participants were being treated by a physiotherapist. The other two participants had experienced physiotherapy treatment in the past. All participants consulted other healthcare professionals (e.g., general practitioners, specialists (orthopaedists, neurologists, rheumatologists, rehabilitation physicians, anaesthesiologists), psychologists, occupational therapists, osteopaths).

**Table 1:** Patient’s characteristics

Code	Sex	Age (Y)	Onset*	Duration of pain (Months)	History of pain	Pain intensity (0–10 NPRS)	Disability (0–50 NDI)	Fear of activities (0-100 NeckPix)	Patient Specific Functional Scale (0–10 PSFS)
P1	f	50	G	3	Yes	7	20	5	Sleeping (7) Doing office work (9) Reading (6)
P2	f	53	G	444	Yes	9	25	35	Sleeping (8) Driving a car (7) Reading (8)
P3	f	55	S - T (CA)	18	Yes	2	26	65	Doing sports (8) Doing housework (8)
P4	f	61	S - NT	408	No	8	17	71	Brushing the dog (9) Providing power with arms (8) Vacuuming & Ironing (8)
P5	m	36	S - T (CA)	108	No	7	31	54	Looking up (8) Lifting (8) Reading (6)
P6	f	52	G	6	Yes	4	15	25	Sitting (5) Walking (long distances) (3) Lifting (5)
P7	f	51	G	414	Yes	3	22	5	Intensive listening (6) Turning (head) in bed (6) Working at a monitor (4)
P8	f	48	S - NT	24	Yes	7	30	93	Lifting (10) Hanging the laundry (7) Vacuuming (7)
P9	f	46	S- NT	60	Yes	7	20	43	Walking (long distances) (7) Doing sports (6)
P10	m	52	S - T (F)	4	No	6	20	66	Working at a monitor (6) Cycling (7) Driving a car (7)
P11	m	43	G	6	Yes	5	15	49	Sleeping prone (10)
P12	f	26	G	120	Yes	7	15	64	Lifting a baby (8) Carrying shopping bags (9)
P13	f	77	S - T (F)	3	Yes	6	16	17	Lifting my arms (8) Carrying shopping bags (8) Driving a car (8)
P14	f	48	S - T (CA)	13	Yes	7	31	27	Getting out of bed (7) Driving a car (10) Walking, cycling (9)
P15	f	45	G	15	Yes	4	16	0	Playing with kids (3) Doing household activities (5) Sleeping (3)
P16	f	54	S - T (CA)	300	Yes	7	32	0	Walking (5) Sitting/ cycling (7) Lifting (5)
P17	m	34	S - T (CA)	11	No	4	19	.	Working at a monitor (3) Practicing martial arts (10) Renovating (8)
P18	f	35	S - T (CA)	84	No	6	25	0	Reading (6) Writing (6) Doing household activities (8)
P19	f	23	G	5	No	4	15	40	Lifting (7) Sitting (9) Exercising with arms (6)
P20	m	46	G	30	No	5	25	31	Driving a car (6) Doing sports (4) Working (7)

* G: Gradual or S: Sudden, and if sudden; T: Traumatic (by Car Accident (CA) or fall (F)) or NT: Non- Traumatic; f: female; m: male; Y: year; NPRS: Nummeric Pain Rating Scale; NDI: Neck Disability Index, 15–24 points moderate disability (*N* = 12), 25–34 points severe disability (*N* = 8)

### Interview characteristics

Eighteen interviews were conducted face to face (6 at home and 12 at the clinic) and 2 interviews were conducted via MS Teams. Each interview lasted between 35 and 50 min.

### Perceptions of people with chronic disabling non-specific neck pain

A range of perceptions were present in the narratives of people with neck pain. Five themes demonstrating these perceptions emerged, being; 1) ‘How my neck pain journey began and why it continued’, 2) ‘Labelling my condition’, 3) ‘Impact: Multiple symptoms that require attention and action’, 4) ‘Coping with neck pain’ and 5) ‘Along the road: perceptions and experiences’. Each theme was subdivided in one or two subthemes to further specify the perceptions. The themes and subthemes are presented in Table 2 and explained below (see also Supplementary file 2 for an extended version of Table 2, including quotes).

**Table 2:** Perceptions present within the narratives of people with chronic disabling non-specific neck pain

Themes	Subthemes	Codes		Best fit with perception dimension…
How my neck pain journey began and why it continued	Uni- versus multicausal contributing factors	A particular event		Causes
		A combination of multiple causal factors		
	Maintaining factors	An accumulation of multiple factors and/or a vicious circle		Provoking factors
Labelling my condition	A range of beliefs; from unknown to clear (predominantly biomedical) beliefs	Unknown		Identity
		Stress, dissatisfaction or being vulnerable		
		Anatomical/ pathophysiological substrate		
Impact: Multiple symptoms that require attention and action	The impact of neck pain on daily functioning	Just keep going		Consequences
		Withdrawal from activities		
	Emotional impact	Feeling insecure, frustrated, guilty, lonely, worrying		Emotional representation
		Difficult to accept		
Coping with neck pain	Choosing the coping strategies that seem to make sense	Limit the load		Controllability
		Building strength and resilience		
		Regaining mobility		
		Keep moving		
		Being meaningful and having some distraction		
Along the road: perceptions and experiences	Uncertainty for the future	Optimistic, hopeful		Timeline & emotional representation
		Pessimistic		
		Uncertain		
	Need for an appropriate explanatory construct	A(n) (endless) quest		Coherence

#### Theme 1: how my neck pain journey began and why it continued

Perceptions regarding causal factors differed between people with a sudden (*N* = 11) and a gradual (*N* = 9) onset of their pain. The subtheme was labelled as ‘uni- versus multicausal contributing factors’. The majority of the participants who reported a sudden onset related the cause of their neck pain to a particular event, such as a car accident (P3, P5, P14, P16, P17, P18), a fall (P10, P13) or an epidural injection before delivery (P4). Two participants stated they ‘suddenly woke up with it’ (P8, P9).

*Q1: 21 Years ago I had a serious car accident which resulted in a whiplash. Since then I have neck pain, sometimes on a daily basis, sometimes with pain-free periods in between. (P15*)

When the episode of neck pain started gradually, the neck pain was attributed to a combination of work exposures, incorrect posture and/or the inability to relax the neck-shoulder region (P1, P2, P4, P6, P7, P9, P12, P19, P20), stress and a lack of relaxation (P1, P6, P7, P11, P12, P15, P20), aging or deconditioning (P1, P4, P9, P14, P20) and/or underlying anatomical disorders (e.g., arthrosis or scoliosis (P2, P4, P7, P19), surgery in another region (P2), or a hearing problem (P7)). For example,

*Q2: I think it is an accumulation of different factors…not having a good time during my internship…, sitting behind my screen for a long time, an incorrect posture, a wrong chair and bed, overload… (P12)* or

*Q3: My age will be of influence. I am a bit older, I’ve been physically active and as a result I have these complaints. My parents are in a similar situation, my mother is diagnosed with a hernia… So yes, old age is coming, my body has had a lot to endure over the years. (P09)* or

*Q4: I think because of stress. The past 40 years were stressful to me and I think that this had consequences for my body. (P11)*

All participants indicated that the persistence of their pain was the result of multiple factors (subtheme ‘maintaining factors’). Many participants indicated that ‘things accumulated’ (P1, P3, P4, P7, P9, P12, P15, P19, P20) (as reported in Q2) or they had ended up in a vicious circle (P2, P3, P10, P14). They stated that they felt trapped by a multifactorial condition, which was difficult to get out of. For example,

Q5: I’m not a good sleeper and in combination with my neck pain and headache….that in turn affects how rested I am, and influences my concentration, it’s a vicious circle…I’ve a busy job, it’s hard to dose my load, that’s really difficult. (P10)

Mechanical loading factors were reported as maintaining factors, such as incorrect posture, and engaging in certain physical activities, such as lifting or cycling. For example,

*Q6: I look a lot at a computer screen and I think my posture is incorrect, I am also a fanatic cyclist and of course that is not good for your neck. (P10)* or

*Q7: I lifted a lot of heavy things, constantly worked with my arms. In my work my posture was the same every day… that obviously affects your neck. (P09)*.

Others considered that their ongoing condition was related to reduced physical capacity as a consequence of a low activity level, ageing, previous surgery and/or unresolved tissue damage related to a mechanical trauma in their history. For example,

*Q8: I had a car accident last year… they call it a whiplash; your muscles get damaged. Generally, that should be healed in half a year, but that is not the case with me…, I think that’s because I was operated on my stomach a few times, so I wasn’t physically fit, and yes, I’m also almost 50, so that will also play a role. (P14)*

Some reported the contribution of psychological factors, such as stress, uncertainty, anxiety and a lack of distraction or meaningful activities. For example,

*Q9: Whenever I turned my head, my neck made a cracking sound and I thought ‘this can’t be right’… I became afraid to turn my head and tried to move my neck as little as possible… I ended up losing my job. I became depressed because of this; I suffer from neck pain all the time and I really miss my job because I hardly have any social contact anymore… I couldn’t cycle or walk anymore because of my neck pain, while I always enjoyed these activities. The lack of distraction made me eat and smoke a lot… I thought ‘it probably won’t get any better’…If I always have to live like this, I’d rather die*. *(P3)*

Others related their persistent neck pain condition to external factors, such as their bed, pillow or weather conditions.

#### Theme 2: labelling my condition

The subtheme regarding the labelling of their condition was ‘a range of beliefs; from unknown to clear (predominantly biomedical) beliefs’. For five participants it was difficult to label their condition, they ‘didn’t know’ (P5, P8, P12, P16, P20). For example,

*Q10: I don’t know where the pain comes from, they [the doctors] say ‘it is because of a hernia’, but I don’t have an explanation for it myself. (P8)*

Others used more general terms to describe their condition, such as ‘being vulnerable’ (P3, P13, P15), ‘nothing serious’ (P12), ‘a result of stress or dissatisfaction’ (P3, P6, P11, P15). For example,

*Q11: There is also a lot going on mentally…, most of it is in my head. (P3)*

Most participants linked their pain to an underlying anatomical or pathophysiological mechanism. The presence of increased muscle tension, muscle cramps, degenerative processes (e.g., arthrosis), nerve entrapment or disc herniation, malalignment of a joint and/or instability of the spine was associated with their neck pain. For example,

*              “Q12: My scoliosis and my pain, that’s a 100% match (P19)* or

*Q13: What do these cracking noises mean to me? Uhh… maybe something is wrong … that some bones are not in the right position? (P13)”*.

#### Theme 3: impact: multiple symptoms that require attention and action

Persistent neck pain was often accompanied by other symptoms, such as reduced ability to concentrate, headache, dizziness, thoracic and/or shoulder pain, clicking/crepitus, being more sensitive to other stimuli, fatigue, and visual and sleep disturbances. For example,

*Q14: Pain and feeling nauseous and very, very tired, but also not being able to sleep, that is really annoying…sometimes when I would turn my neck too far, I can’t look through my eyes because of a headache. (P2)*

These symptoms affected the participants’ daily functioning (subtheme: ‘the impact of neck pain on daily functioning’). Several participants were hindered in their normal activities, but ‘just kept going’ (P1, P4, P10, P12, P19), while others felt severely disabled and compelled to pace themselves, limit or even stop their (social) activities, their work and/or sports (subtheme ‘impact on daily functioning’). This sometimes made their situation unbearable (P2, P3, P5, P8, P14, P16, P17). For example,

*Q15: I have had this pain for over 10 years but, nevertheless, I still just do everything. (P12)* or

*Q16: Anything I do, I feel pressure in my neck. It doesn’t matter what I do, even when driving I have to stop after half an hour because the pain starts again. I can’t do anything anymore. (P5)*.

In all participants the complex of symptoms and its perceived consequences affected them emotionally (subtheme ‘emotional impact’). Pain was reported to be difficult to cope with (as reported in Q9, Q5) and participants often felt frustrated, angry, insecure, anxious, dejected, sad or depressed. For all participants the pain constantly required attention,which made it difficult to accept the situation and was a facilitator for seeking care. For example,

*Q17: Sometimes I just want to cry, then I don’t want to see anyone, I want a solution. I just keep searching…, because I can’t live like this. (P8)*

Some felt guilty about the impact of their pain on their loved ones (P2, P3, P5, P8, P17, P12), sometimes withdrew and felt lonely (P3, P7, P8, P14). For example,

*Q18: Interviewer: Does your situation affect your mood? Participant: Yes, for example, when my family wants to do something and I am in a lot of pain, I can’t join them. Or they want to invite someone, then… if you’re in pain you don’t want to see anyone, that hurts me a lot [gets emotional]. I just want to participate…, join in with my children, with my family, with my husband, yes… just like before, doing everything myself. (P8)*

#### Theme 4: coping with neck pain

The way participants made sense of their pain guided their health behaviour (subtheme: ‘choosing the coping strategies that seem to make sense’). Those who perceived their neck was ‘vulnerable’, tended to limit their load and/or avoided more strenuous activities (such as lifting, cleaning windows, prolonged computer work, reading), withdrew from social and/or work-related activities, adapted the context (e.g., changed their chair or bed) and/or sought social support (children or partner). For example,

*Q19: I always have to think ahead, sometimes my husband accompanies me to assist me, otherwise it’s too heavy for me. Sometimes others think ‘oh, let’s go out for dinner’, but I can’t work on a photography assignment and go out for dinner afterwards. (P2)*

If they perceived their neck was ‘worn out’, they believed this process would continue and this should be accepted. For those who perceived that de-conditioning played a role in their pain, their strategy was building strength and resilience. For example,

Q*20: In the beginning I was totally out of shape, but now I really notice that I am getting stronger. (P3)*

Others perceived their neck was ‘stuck’ and thought that ‘it should be loosened’. These people used multiple parallel strategies, such as general movements, exercises, stretching, massage, medication, heat and/or paying attention to their posture. For example,

*Q21: It is completely tense, so I try to stretch the other side and I use some massage lotion. (P4)*

All participants indicated they benefited from exercise, although the duration, frequency and intensity of the exercises varied enormously. For example,

*Q22: Sometimes there are weeks with quite a lot of office work, then I really have to walk in between or play table tennis or something…, in other weeks I cycle a lot, that’s better… When exercising [running], I have pain in the beginning, but once I am warmed up, the pain becomes less. I know that, so I keep running (P1)* or

*Q23: In my case, walking means strolling and I cycle very slowly, otherwise it becomes too much,… at least it’s good for me. (P11)*.

Many participants mentioned that having a job was very important for them. Work provided distraction, positive energy, satisfaction and made them meaningful to others. For example,

*Q24: I don’t want to stay at home because of my pain, that only makes me more depressed. I have to carry on, I need the distraction, I help a child [at my workplace; a primary school], for example, that helps me enormously, that’s how I keep my head above water. (P9*)

Sometimes work was also perceived to be burdensome because ‘too much is asked’ (P3, P5, P7, P16, P17). Loss of work was accompanied by strong negative emotions (as reported in Q9), although it was sometimes also a relief to have less obligations (P7, P16, P17).

*Q25: Unfortunately I can’t fulfil my job anymore, but as a result of this there is more time for myself…I can now set my own limits and have less obligations. It sounds very simple, but it is very important to me. (P16)*

#### Theme 5: along the road: perceptions and experiences

Some participants’ perceptions were clearly expressed, consistent and seemed strongly engrained, while others used multiple explanations, which were less clearly defined, and/or were still looking for a plausible explanation for their pain and how to cope with it. The participant’s expectations regarding the prognosis differed and were prompted by the duration of their neck pain episode, previous experiences, treatment results and their beliefs about the underlying (pathophysiological) process. Many participants remained hopeful and continued to look for a solution and (partial) relief from their symptoms by visiting health professionals and adapting their behaviour (P1,P2,P3,P6,P8,P10,P11,P12,P13,P15,P18,P20). Many of them did not believe that their pain would disappear completely, but expected that their situation would be bearable and that they could cope with it (P1,P3,P6,P11,P13,P15,P18,P19,P20). For example,

*Q26: In 10 years time, I don’t think I will be without neck pain, but it would be nice, well I would like it, if I can create a situation, together with my physiotherapist, that I still have some neck pain, and that I have the right exercises, so when the pain comes I know what I can do about it. (P15)*

Others were more negative (P4,P5,P7,P8,P14,P16) about the prognosis. For example,

*Q27: What I can do physically has been reduced considerably, and that makes me anxious; where will this process end? (P16)*

Uncertainty about the future amplified the impact of the participants’ neck pain. This uncertainty was sometimes related to everyday situations, such as fear of pain when resuming activities, but mainly concerned the long-term perspective; some participants felt uncertain whether their condition would improve (P5, P8, P9, P10, P12, P14, P16, P17) (subtheme ‘uncertainty for the future’). For example,

*Q28: Yes, I’m really worried about that: ‘will it ever completely go away or will I always have symptoms?’, that is my main concern. (P10)*

Most participants indicated that it was important for them to have an appropriate understanding of their condition that fit their situation and that would help them to gain control over it (subtheme ‘need for an appropriate explanatory construct’). For example,

*Q29: I try to understand what’s going on and when, at some point, you make sense of it, then it is easier for me to cope with it. (P16)*

Frequently, they described a path of trial and error accompanied by negative emotions and feelings of powerlessness. This was especially the case in participants with severely disabling neck pain, who lacked positive experiences with (self)management of their pain and who visited multiple healthcare professionals. For example,

*Q30: I really don’t know. I visit the hospital or my doctor so often, but I have no solution, neither do they…I’ve already had a lot of physiotherapy. and I consulted the pain clinic, I received [Buprenorphine] patches and laser therapy., I frequently visited a psychologist, but that didn’t work for me. Maybe I need another treatment, another diagnostic assessment. Yes, now I am waiting, because the 22nd I have to go to the doctor again, I’m desperate… I will visit my country of birth to go to a doctor, to have an examination there and see what they say. (P8*)

In participants with a gradual onset of pain and a broad biopsychosocial view, who had experienced that they could influence their condition themselves, beliefs seemed more flexible and optimistic (as reported in Q26).

Most participants seemed to be open to different coping strategies, as long as these strategies fit with the way they made sense of their neck pain or were logical to them. All participants had a perception that ‘a quick fix solution’ was not realistic. For example,

*Q31: It’s not a broken leg that you can see and repair. Unfortunately, I have experienced that this cannot be fixed. So, I understand that it is not easy for a healthcare provider… At the moment, I do experience that my situation is being looked at from a broader perspective, and that supports me. (P16)*

#### Relationships between the themes

The participants’ narratives showed a strong relationship between the themes. The way participants labelled their condition influenced their coping strategies. These strategies were then adjusted based on own experiences and/or information from others. Whether or not the actual experience matched the participants’ expectations was a strong determinant of their confidence to feel in control in the future. Uncertainty about prognosis, prompted by negative experiences in the past, was a major stressor.

### The formation of illness perceptions

Three themes emerged regarding the formation of illness perceptions, being 1) ‘A dominant role of healthcare professionals’, 2) ‘Combining the patient’s and the clinician’s perceptions’ and 3) ‘The importance of (ex)changing perspectives’. The origin, themes and subthemes are presented in Tables 3 and explained in this paragraph (see also Supplementary file 3 for an extended version of this Table, including quotes).

**Table 3:** The formation of the perceptions of people with chronic non-specific neck pain

Origin	Theme	Subtheme
Professionals	Dominant role of healthcare professionals	Biomedically orientated information
Family		
Own Experiences		
Engagement with healthcare professionals	Combining the patient’s and the clinician’s perceptions: Searching for mutual understanding	
	The importance of (ex)changing perspectives	

#### Theme 1: a dominant role of healthcare professionals

Although personal experiences were important in the origination of illness perceptions (see below Q34), many of the participants described their illness perceptions arose from, or were supported by, information from others (e.g., clinicians, family), with a dominant role for healthcare professionals (Q32,Q35-37). For example,

*Q32: I’ve been to the chiropractor before… he told me that I have some kind of scoliosis (P19)* or

*Q33: Also, my mother said: it’s really hard. You just need a good massage… what my mother always notices is that I watched TV with my head tilted… and I still do this. My partner literally says: ‘head straight’. (P12)* or

*Q34: Interviewer: What made you decide to take more rest? Participant: When I was on holidays for two weeks, I immediately noticed that I was getting better… I didn’t need any medication, and then you go back to work and the symptoms come back, so I thought ‘that’s it’… I also got dizzy when I was on my race bike, so I thought ‘that just isn’t right’, so um.., experiences have taught me that. (P10)*

Several participants indicated that they were overloaded with (sometimes contradictory and often incomprehensible) biomedical information (P4, P9, P7, P12) (subtheme ‘biomedically orientated information’). For example,

*Q35: Some tell me ‘it’s a herniated disc’, based on the information from a CT-scan, and others say ‘it is just a muscle’, so to be honest, I really don’t know what to believe anymore,… I am also diagnosed as having osteoarthritis,… that’s how you get put in a certain box. (P9)*

Some participants initially adopted a biopsychosocial perspective themselves, which seemed to evolve towards a more biomedical perspective due to the influence of interactions with healthcare professionals. For example,

*Q36: So, when the neck pain started I thought; ‘I am young, it won’t be anything serious… I sit here all day [in the office for my internship] and I am not having a good time, this manifests itself in my body’… now [after I visited a physiotherapist] I think I am in pain because of my posture, I am told that I was sitting in an incorrect position on a bad chair. And uhh… my back wasn’t straight and my shoulders weren’t aligned. That’s what my physiotherapist told me…’ (P12).*

Healthcare professionals often emphasized the underlying patho-anatomical or biomechanical processes, and the therapeutic approach was predominantly biomedical in nature. Information from imaging sometimes enhanced the biomedical perspective (as reported in Q35), but could also rule out the influence of biomedical factors, which could be reassuring. For example,

*Q37: …scans were made and then they saw that everything was fine. (P3)*

#### Theme 2: combining the patient’s and the clinician’s perceptions: searching for mutual understanding

Frequently, the participants’ perceptions were a result of the reflection on personal experiences interacting with information from healthcare professionals. Patients and healthcare professionals searched for mutual understanding of the patient’s condition. Sometimes the participants’ perceptions were in line with the perceptions of the healthcare professional and these participants mainly needed confirmation and support (P1,P4,P6, P7, P11,P13,P16, P19). For example,

*Q38: The therapist said ‘let’s start with relaxed movements’, … she also told me that a painkiller would be released., which was enough for me to start with exercising. Interviewer: Were you comfortable with this strategy? Participant: Yes, …I try to move without using extra forces, so that you just get your musculoskeletal system a little more flexible. And I think that it’s important to do this, as much as possible. (P11)*.

Others needed a different perspective to break the vicious circle they were in (P2,P3,P10,P15,P18,P20). For example,

*Q39: I’m glad I visit someone who opened my eyes,… I’m moving my head more frequently, it is painful, but he [the therapist] says:‘nothing will happen to your neck’, so I now realise that I just have to exercise, cycle, walk and keep going and I feel that my condition improves. (P3*)

Sometimes the explanation provided by their healthcare professional was not aligned with the participant’s perceptions of their condition, which made it more difficult to cope with their pain. Some participants indicated that they had met clinicians who assumed that their health problem could (and should) be resolved, while (based on their own experiences) they no longer had this expectation (P1, P9, P16, P18, P17). For example,

*Q40: He told me that with this approach, it should get better,. he suggested: ‘if you do this and that, then it should get better’, but in my situation this is not the case,… I feel that I’m falling short, that it is my fault… One day I’m feeling really bad and the next day it’s better, but that doesn’t mean that everything can be resolved, that’s just not true. At least, that’s what I have experienced. (P16)*

Subsequently, when the course of their condition deviated from the scenario outlined by their healthcare professional, several participants felt guilty (“Is the persistence of pain my fault?”), and became insecure, despondent or frustrated. They lacked someone who paid attention to their situation and who continued to partner with them to improve it. For example,

*Q41: I just need someone to say: ‘how are you today? How was your week?’ And not that the message is: ‘if you do this or do that, then next week it will be much better… [I’d appreciate it] if it is okay that it [the pain] is there. Perhaps the therapy should be: how can I support you to function optimally despite the pain? (P16)*

#### Theme 3: the importance of (ex)changing perspectives

Most participants indicated that it was important for them to exchange perspectives with the professional. They noticed that it was helpful to them if clinicians listened carefully, validated their feelings and thoughts, and shared ideas regarding perceptions relating to their condition and strategies to cope with it. According to some, gaining insight into their own condition was considered an important part of the solution (P2,P3, P16, P18, P20). For example,

*Q42: What does the explanation mean to me? It helps me a little bit in understanding my own body…and then, usually the next time, I feel much, much better. (P20)*

## Discussion

This study represented a comprehensive exploration of illness perceptions of people with disabling chronic neck pain using the Common Sense Model. All participants reported that they felt trapped within a complex multifactorial problem that continuously required attention and action and was difficult to get out of. Although the participants were open to associate multiple biopsychosocial factors with the persistence of their neck pain, a biomedical paradigm was dominant with regard to the labelling of their condition and their coping strategy. Five coping strategies were identified, being ‘limiting the load’, ‘building strength and resilience’, ‘regaining mobility’, ‘keep moving’ and ‘being meaningful’. How people understood their condition was strongly related to their way of coping. Information from healthcare professionals strongly influenced how people tried to understand their pain. Participants underlined the importance of mutual understanding and exchanging perspectives in the engagement with health professionals, resulting in an explanatory model that makes sense to them and supports them in self-managing their situation.

The results from this study are consistent with the themes identified in other qualitative studies in people with neck pain, being ‘a condition of multiple symptoms which is difficult to cope with’ [10, 26, 36], ‘a continuous search for the appropriate strategy to influence pain’ [26, 27, 29] and ‘movement behaviour’ [10, 28, 36]. Illustrative of this, some participants in the current study indicated that as time progressed they understood their condition differently (e.g., they learned that despite cracking sounds, it was safe to move) and developed different coping strategies (e.g., they experienced that they could influence their condition by relaxing and moving). For others this was still a quest and a process of negative experiences, frustration and uncertainty. Regarding the theme ‘expectations of recovery*’* [10, 27] as identified in other studies, some participants in the current study reported that thay had received helpful information and were optimistic about the future (“[the therapist] opened my eyes… so I now realize that I just have to exercise, cycle, walk and keep going and I feel that my condition improves”), while others reported that they still felt uncertain and/or ignorant regarding their condition. They had received conflicting information (e.g., Q35) or information that did not fit their own experiences, which did not help to gain control over their situation. In addition, other studies also found that people associated multiple biopsychosocial factors with the onset or persistence of their neck pain [37, 70]. In people with WAD, the influence of compensation and funding systems on obtaining optimal care to improve their condition was explicitly mentioned [27], while in the current study only one participant (out of eight people with traumatic neck pain and twelve people with non-traumatic neck pain) raised this as a concern. It might be that this reflects the difference between a person seeking financial compensation for injury versus someone who is experiencing pain where there is no one at fault legally and/ or that the financial aspect no longer played a role in the other participants with traumatic neck pain. Also, differences in the compensation systems between countries might play a role. Furthermore, a study in people with work-related neck and arm pain and WAD, that used a questionnaire to evaluate people’s beliefs, found that people had ‘a desire to be fixed’ [28], while most participants in the current study indicated that they considered this unrealistic. This difference might be explained by the level of chronicity in participants in the current study and their experiences of only a short term relief of passive interventions (“I think I’ve seen a physiotherapist or 10 and they all said ‘oh let me take a look’, …. they told me my vertebrae weren’t aligned and corrected the position of my vertebrae, but that didn’t bring me what I had thought”(P20)).

The finding in the current study that the participants’ perceptions were strongly influenced by healthcare professionals is consistent with other studies in people with WAD [10] and low back pain [24, 71]. In the current study, healthcare professionals often emphasized the underlying patho-anatomical or biomechanical processes and the therapeutic approach was predominantly biomedical in nature. This was also found in other qualitative studies in people with chronic musculoskeletal pain conditions [63, 71, 72]. Such a biomedical focus is at odds with a contemporary biopsychosocial understanding of neck pain, and does not seem to align with the poor correlation between neck pain and posture [73–75] or degeneration [76–78]. A substantial amount of research shows that clinician’s training and beliefs are often at odds with evidence [79, 80], which may hinder adequate management [4].

In this study, the Common Sense Model was used as a framework for the interviews to explore the illness perceptions of people with neck pain. Although the semi-structured character of the interviews gave space to the individual narrative, all the narratives could be placed within the existing dimensions of the model. However, the term ‘maintaining factors’ seemed more appropriate for the narratives of this chronic neck pain population than the term ‘provoking factors’ as proposed by Wilgen et al. (2014). The emotional impact reported by the participants underlines the importance of the dimension ‘emotional representations’, that was later added to the Common Sense Model [22].

We believe the narratives to be representative of people with chronic disabling neck pain who visit healthcare professionals. We recruited 20 participants with chronic disabling neck pain from six different practices by fourteen physiotherapists and obtained a wide range of different illness perceptions. Theoretical saturation was reached and we believe that the selection bias within this study is limited, as all but one of the participants who met the inclusion criteria and who were asked to participate (using purposive sampling), were willing to participate in the study. In addition, all interviews were conducted by an experienced interviewer, the data were member checked, coding of the first eight interviews was independently checked by another researcher to ensure the findings reflected the data and ongoing analysis was repeatedly discussed in the research team. As a result of the recruitment strategy, most participants were involved in physiotherapy interventions (18/20) or had recent experiences with physiotherapy (2/20). This provided an opportunity to gain insights how their initial ‘lay’ perceptions were influenced by therapist-informed perceptions. However, attending a physiotherapist might have affected the participants’ narratives, so the transferability of the results to those who did not seek care at all, or to those who sought only medical care, is unknown. In addition, it should be noted that although the sample was diverse in terms of age, gender, duration of the neck pain episode, and social and cultural background (e.g. urban/regional, with/without migration background), we were unable to create subgroups due to the size of the sample.

### Implications for clinical practice

Exploring patient’s illness perceptions provides a deep understanding of the labelling of their condition, as well as their coping and emotional responses. This can enhance person-centred care as participants indicated it was important for them to be able to share their feelings and thoughts, understand their condition, and validate their coping strategies. There is emerging evidence that addressing illness perceptions may facilitate patient recovery [4, 81–84]. Although participants mainly reported biomedically oriented illness perceptions regarding the labelling of their condition and their coping strategy, they frequently used a broad biopsychosocial perspective to describe the maintaining factors of their pain. This suggests that their illness perceptions are not so strongly engrained as to be unchangeable [16, 85]. We consider this as an opportunity for healthcare professionals to explore the patient’s initial ‘lay’ perceptions, facilitate coping strategies that take into account both physical as well as psychosocial factors and encourage patients to be more actively involved in the development of effective management strategies. Information from healthcare professionals strongly influences a persons’ illness perceptions, therefore clinicians should truly embrace the biopsychosocial paradigm. However, many physiotherapists feel unskilled and unprepared to explore and address psychosocial factors in patients with chronic pain [79, 86, 87]. Extensive, individualized and supervised training in influencing illness perceptions, within the context that is meaningful for the clinician and using evidence based strategies, is required to enhance these skills [67, 80, 88]. Furthermore, the provision of consistent (interdisciplinary) information, appears to be a precondition for adequate management [89].

### Electronic supplementary material

Below is the link to the electronic supplementary material.

Supplementary Material 1

## Data Availability

The data (i.e., the demographic and neck pain related data of the participants) are available through DataverseNL: 10.34894/MYCFIY on reasonable request.
