# Supplementary material for: Illness perceptions in people with chronic and disabling non-specific neck pain seeking primary healthcare: a qualitative study
Source: BMC Musculoskelet Disord. 2024 Feb 27;25:179. doi: 10.1186/s12891-024-07302-7 (PMC10900625; doi:10.1186/s12891-024-07302-7)
Supplement: Supplementary file 1 — Supplementary Material 1 [file 12891_2024_7302_MOESM1_ESM.docx]

**Supplementary file 1. Conversation guide**

| Topic | Main question | Sub questions + emotional representation and coherence |
| --- | --- | --- |
| Identity | How do you label your condition?  Why do you think this? | Can you explain me what this means to you?  How do you feel about it? |
|  |  | Did you get a diagnose?  Can you explain to me (in your own words) what this diagnose meant to you?  How much does this diagnose make sense to you?  How do you feel about it? |
|  |  | Do you have previous experiences with this kind of pain? |
|  |  | Have scans been made?  What did they show?  What do you think of that?  What does that mean to you? |
|  |  | Do you have other ideas regarding your condition?  Why do you think this? |
|  |  | Have you received information regarding your condition from others?  Has this information been contradictory to other information?  In which way?  What did this mean to you?  How did/do you feel about it? |
| Causes | Which factors, do you think, are responsible for causing your neck pain?  Why do you think this? | How important are these factors? |
|  |  | Are there other factors that contribute to your pain experience? Why do you think this? |
| Provoking factors |  |  |
|  | Which factors influence the persistence of your neck pain? Why do you think this? | Have you considered other factors? Which? Why? |
| Consequences | What consequences does your neck pain have to your personal (quality of) life (both physically and socially)? | How does your neck pain affect your functional capacity / work / private life / social life / sports / etc.? |
|  |  | How does your neck pain affect your movement behavior?  In the questionnaire you mentioned that you feel disabled in …., can you explain this?  Can you show me this activity?  What happens when you perform this activity? |
|  |  | What does this mean to you? How do you feel about it? |
|  |  | Are there activities that you perform/ avoid because of your pain? Why?  How do you feel about that?  Can you show me these activities?  What happens when you perform these activities? |
|  |  | What are your expectations regarding consequences in the near future? |
| Timeline | How long do you think this period with neck pain might last? Why do you think this? | What does this mean to you?  How do you feel about it?  How positive / negative are you regarding the prognosis? |
| Controllability | Can your condition be cured? How?  Can you or others keep the pain under control? | How do you cope with it?  What can you do to make yourself feel better about it? Why do you think this will help you?  Does it work? |
|  |  | Are there other things you can do to regain control about your pain?  Does it work? |
|  |  | What does it mean to you that you can / can’t control your pain?  How does this affect you? |
|  |  | Do you need others (partner, family, employer, friends, care giver)?  Who? What for? |
|  |  | What do you expect of care givers?  Why? |
|  |  | What does it mean to you that others can / can’t control your pain?  How do you feel about that? |
|  |  | What was your *expectation* regarding the support of the care giver? |
|  |  | What *actually was* the role of the care giver (physiotherapist or general practitioner) in regaining control about your pain? |

**Supplementary Table 2. Perceptions present within the narratives of people with chronic disabling non-specific neck pain**

| Themes | Subthemes | Codes | Quotes (Px) |
| --- | --- | --- | --- |
| How my neck pain journey began and why it continued | Uni- versus multicausal contributing factors | A particular event | Q1 ‘21 Years ago I had a serious car accident which resulted in a whiplash. Since then I have neck pain, sometimes on a daily basis, sometimes with pain-free periods in between.’ (P15) |
|  |  | A combination of multiple causal factors | Q2 ‘I think it is an accumulation of different factors…not having a good time during my internship…, sitting behind my screen for a long time, an incorrect posture, a wrong chair and bed.., overload…’ (P12)  Q3 ‘My age will be of influence. I am a bit older, I’ve been physically active and as a result I have these complaints. My parents are in a similar situation, my mother is diagnosed with a hernia… So yes, old age is coming, my body has had a lot to endure over the years.’ (P09)  Q4 I think because of stress. The past 40 years were stressful to me and I think that this had consequences for my body. (P11) |
|  | Maintaining factors | An accumulation of multiple factors and/or a vicious circle | Q5 ‘I'm not a good sleeper and in combination with my neck pain and headache....that in turn affects how rested I am and influences my concentration, it's a vicious circle…I’ve a busy job, it’s hard to dose my load, that’s really difficult.’ (P10)  Q6 ‘I look a lot at a computer screen and I think my posture is incorrect, I am also a fanatic cyclist and of course that is not good for your neck.’ (P10)  Q7 ‘I lifted a lot of heavy things, constantly worked with my arms. In my work my posture was the same every day… that obviously affects your neck’. (P09)  Q8 ‘I had a car accident last year… they call it a whiplash; your muscles get damaged. Generally, that should be healed in half a year, but that is not the case with me…, I think that's because I was operated on my stomach a few times, so I wasn't physically fit, and yes, I'm also almost 50, so that will also play a role’ (P14).  Q9 ‘Whenever I turned my head, my neck made a cracking sound and I thought 'this can't be right'… I became afraid to turn my head and tried to move my neck as little as possible… I ended up losing my job. I became depressed because of this; I suffer from neck pain all the time and I really miss my job because I hardly have any social contact anymore... I couldn't cycle or walk anymore because of my neck pain, while I always enjoyed these activities. The lack of distraction made me eat and smoke a lot... I thought ‘it probably won’t get any better’...If I always have to live like this, I'd rather die.’ (P3) |
| Labelling my condition | A range of beliefs; from unknown to clear (predominantly biomedical) beliefs | Unknown | Q10 ‘I don’t know where the pain comes from, they [the doctors] say ‘it is because of a hernia’, but I don’t have an explanation for it myself.’ (P8) |
|  |  | Stress, dissatisfaction or being vulnerable | Q4 (see above)  Q11 ‘There is also a lot going on mentally…, most of it is in my head.’ (P3) |
|  |  | Anatomical/ pathophysiological substrate | Q12 ‘My scoliosis and my pain, that’s a 100% match’ (P19)  Q13 ‘What do these cracking noises mean to me? Uhh… maybe something is wrong … that some bones are not in the right position?’ (P13) |
| Impact: Multiple symptoms that require attention and action | The impact of neck pain on daily functioning | More than just neck pain | Q14 Pain and feeling nauseous and very, very tired, but also not being able to sleep, that is really annoying…sometimes when I would turn my neck too far, I can’t look through my eyes because of a headache. (P2) |
|  |  | Just keep going | Q15 ‘I have had this pain for over 10 years but, nevertheless, I still just do everything.’ (P12) |
|  |  | Withdrawal from activities | Q16 ‘Anything I do, I feel pressure in my neck. It doesn’t matter what I do, even when driving I have to stop after half an hour because the pain starts again. I can’t do anything anymore.’ (P5) |
|  | Emotional impact | Feeling insecure, frustrated, guilty, lonely, worrying | Q9 (see above)  Q18 Interviewer: ‘Does your situation affect your mood?’ Participant: ‘Yes, for example, when my family wants to do something and I am in a lot of pain, I can’t join them. Or they want to invite someone, then… if you're in pain you don't want to see anyone, that hurts me a lot [gets emotional]. I just want to participate…, join in with my children, with my family, with my husband, yes… just like before, doing everything myself’. (P8) |
|  |  | Difficult to accept | Q17 Sometimes I just want to cry, then I don’t want to see anyone, I want a solution. I just keep searching…, because I can’t live like this. (P8) |
| Coping with neck pain | Choosing the coping strategies that seem to make sense | Limit the load | Q19 ‘I always have to think ahead, sometimes my husband accompanies me to assist me, otherwise it’s too heavy for me. Sometimes others think ‘oh, let’s go out for dinner’, but I can’t work on a photography assignment and go out for dinner afterwards’. (P2)  Q25 ‘Unfortunately I can't fulfil my job anymore, but as a result of this there is more time for myself…I can now set my own limits and have less obligations. It sounds very simple, but it is very important to me’. (P16) |
|  |  | Building strength and resilience | Q20 ‘In the beginning I was totally out of shape, but now I really notice that I am getting stronger’. (P3) |
|  |  | Regaining mobility | Q21 ‘It is completely tense, so I try to stretch the other side and I use some massage lotion’. (P4) |
|  |  | Keep moving | Q22 ‘Sometimes there are weeks with quite a lot of office work, then I really have to walk in between or play table tennis or something…, in other weeks I cycle a lot, that's better… When exercising [running], I have pain in the beginning, but once I am warmed up, the pain becomes less. I know that, so I keep running (P1)  Q23 ‘In my case, walking means strolling and I cycle very slowly, otherwise it becomes too much,... at least it's good for me’. (P11) |
|  |  | Being meaningful and having some distraction | Q24 ‘I don’t want to stay at home because of my pain, that only makes me more depressed. I have to carry on, I need the distraction, I help a child [at my workplace; a primary school], for example, that helps me enormously, that’s how I keep my head above water’. (P9) |
| Along the road: perceptions and experiences | Uncertainty for the future | Optimistic, hopeful | Q26 ‘In 10 years’ time, I don’t think I will be without neck pain, but it would be nice, well I would like it, if I can create a situation, together with my physiotherapist, that I still have some neck pain, and that I have the right exercises, so when the pain comes I know wat I can do about it.’ (P15) |
|  |  | Pessimistic | Q27 ‘What I can do physically has been reduced considerably, and that makes me anxious; where will this process end?’ (P16) |
|  |  | Uncertain | Q28 ‘Yes, I’m really worried about that: ‘will it ever completely go away or will I always have symptoms?’, that is my main concern’. (P10) |
|  | Need for an appropriate explanatory construct | A(n) (endless) quest | Q29 ‘I try to understand what’s going on and when, at some point, you make sense of it, then it is easier for me to cope with it.’ (P16)  Q30 ‘I really don’t know. I visit the hospital or my doctor so often, but I have no solution, neither do they…I’ve already had a lot of physiotherapy.. and I consulted the pain clinic, I received [Buprenorphine] patches and laser therapy.., I frequently visited a psychologist, but that didn’t work for me. Maybe I need another treatment, another diagnostic assessment. Yes, now I am waiting, because the 22^nd^ I have to go to the doctor again, I’m desperate… I will visit my country of birth to go to a doctor, to have an examination there and see what they say.’ (P8)  Q31 ‘It's not a broken leg that you can see and repair. Unfortunately, I have experienced that this cannot be fixed. So, I understand that it is not easy for a healthcare provider… At the moment, I do experience that my situation is being looked at from a broader perspective, and that supports me. (P16) |

Qx: Quote number, Px: Participant number

**Supplementary Table 3. The origins of the perceptions of people with chronic non-specific neck pain**

| Origin | Theme-subtheme | Quotes (P..) |
| --- | --- | --- |
| Professionals | Dominant role of healthcare professionals -  Biomedically orientated information | Q32 ‘I’ve been to the chiropractor before…he told me that I have some kind of scoliosis’ (P19)  Q35 ‘Some tell me ‘it’s a herniated disc’, based on the information from a CT-scan and others say ‘it is just a muscle’, so, to be honest, I really don’t know what to believe anymore…, I am also diagnosed as having osteoarthritis,… that’s how you get put in a certain box.’ (P9)  Q37 ‘…scans were made and then they saw that everything was fine’ (P3) |
| Family |  | Q33 ‘Also my mother said: it’s really hard. You just need a good massage… what my mother always notices is that I watched TV with my head tilted… and I still do this. My partner literally says: ‘head straight’.’ (P12) |
| Own Experiences |  | Q34 I: ‘What made you decide to take more rest?’ P: ‘When I was on holidays for two weeks, I immediately noticed that I was getting better… I didn’t need any medication, and then you go back to work and the symptoms come back, so I thought ‘that’s it’… I also got dizzy when I was on my race bike, so I thought ‘that just isn’t right’, so um..., experiences have taught me that. (P10) |
| Engagement with healthcare professionals | Combining the patient’s and the clinician’s perceptions: Searching for mutual understanding | Q36 ‘So, when the neckpain started I thought; ‘I am young... I sit here all day [in the office for my internship] and I am not having a good time, this manifests itself in my body’… … now [after I visited a physiotherapist] I think I am in pain because of my posture, I am told that I was sitting in an incorrect position on a bad chair. And uhh... my back wasn’t straight and my shoulders weren’t aligned. That’s what my physiotherapist told me…’ (P12)  Q38 The therapist said 'let's start with relaxed movements’, ... she also told me that a painkiller would be released.., which was enough for me to start with exercising. Interviewer: Were you comfortable with this strategy? Participant: Yes, …I try to move without using extra forces, so that you just get your musculoskeletal system a little more flexible. And I think that it's important to do this, as much as possible. (P11)  Q39 ‘I’m glad I visit someone who opened my eyes,… I’m moving my head more frequently, it is painful, but he [the therapist] says: ‘nothing will happen to your neck’, so I now realise that I just have to exercise, cycle, walk and keep going and I feel that my condition improves.’ (P3)  Q40 ‘He told me that with this approach, it should get better,… he suggested: ‘if you do this and that, then it should get better’, but in my situation this is not the case,… I feel that I’m falling short, that it is my fault… One day I’m feeling really bad and the next day it’s better, but that doesn’t mean that everything can be resolved, that’s just not true. At least, that’s what I have experienced.’ (P16) |
|  | The importance of (ex)changing perspectives | Q41 ‘I just need someone to say: ‘how are you today? How was your week?’ And not that the message is: 'if you do this or do that, then next week it will be much better… [I’d appreciate it] if it is okay that it [the pain] is there. Perhaps the therapy should be: how can I support you to function optimally despite the pain?’ (P16)  Q42 ‘What does the explanation mean to me? It helps me a little bit in understanding my own body…and then, usually the next time, I feel much, much better.’ (P20) |

Qx: Quote number, Px: Participant number, P: Participant, I: Interviewer
